# Biochemical markers of bone turnover in benign paroxysmal positional vertigo

**DOI:** 10.1371/journal.pone.0176011

**Published:** 2017-05-03

**Authors:** Sun Bin Lee, Chang Ho Lee, Young Ju Kim, Hyoung-Mi Kim

**Affiliations:** Otorhinolaryngology Department, CHA University, Seongnam, Korea; University of Connecticut Health Center, UNITED STATES

## Abstract

**Objective:**

Several studies have suggested a possible relationship between recurrent benign paroxysmal positional vertigo (BPPV) and altered calcium homeostasis in the endolymph of the inner ear. The present study aimed to evaluate the association between Ca^2+^ and vitamin D status and BPPV occurrence as well as the status of bone biochemical markers in osteoporotic patients who were diagnosed with idiopathic BPPV.

**Methods:**

The study included total 132 patients who were referred to our clinic between August 2008 and October 2013. Based on the bone mineral density (BMD) results, the subjects were divided into three groups: normal BMD (n = 34), osteopenia (n = 40) and osteoporosis (n = 58). The biochemical markers of bone turnover including serum Carboxy-terminal telopeptide of type I collagen (s-CTX), osteocalcin, alkaline phosphatase (ALP) and urinary free deoxypyridinoline (u-DPD), were analyzed, along with the serum Ca^2+^ and vitamin D levels.

**Results:**

The mean serum calcium, phosphate and creatinine clearance levels were within the standard laboratory reference range. The incidence of vitamin D deficiency was 11.8% (4/34) in the normal BMD group, 15% (6/40) in the osteopenia group and 43.1% (25/58) in the osteoporosis group. There was a positive correlation between the 25(OH)D and BMD results in the patients with BPPV. Among the bone turnover markers, the osteocalcin and u-DPD levels were significantly elevated in the osteoporotic patients with BPPV. Multiple logistic regression analyses showed that osteoporosis and vitamin D deficiency were associated with BPPV.

**Conclusion:**

Our findings suggest that the prevalence of BPPV in osteoporotic patients is associated with vitamin D deficiency and high bone turnover rates at systemic level, which could disturb local Ca^2+^ homeostasis in the inner ear.

## Introduction

Benign paroxysmal positional vertigo (BPPV), which is the most common cause of recurrent vertigo is characterized by transient vertigo attacks elicited after quick head position changes in a certain direction [1]. Currently, the mechanism of BPPV is explained by displacement of otoconia from the utricle into the semicircular canals. Although various predisposing factors that damage and dislodge otoconia such as head trauma and labyrinthitis have been associated with the development of BPPV, the etiology of BPPV remains undetermined in 50~70% of cases [2, 3]. BPPV can occur at any age, however, a high incidence of BPPV has been documented in elderly patients [2]. The one-year prevalence of BPPV was seven times higher in individuals older than 60 years of age, than in individuals 18 to 39 years of age [4]. An estimated 9~11% of adults over 70 years of age have been diagnosed with BPPV, and unrecognized BPPV is common in geriatric populations with associated morbidity and risk of falls [5]. It is assumed that the high incidence of BPPV in elderly subjects is associated with age-related degenerative changes in the balance system of the inner ear [6], which could be partially accelerated by metabolic bone diseases, such as osteoporosis.

Several studies have suggested a possible relationship between idiopathic BPPV and altered calcium homeostasis in the endolymph of the inner ear. Osteoporosis which is characterized by reduced bone mass and increased bone turnover, occurred more frequently in middle-aged and elderly women suffering recurrent idiopathic BPPV [7, 8], and larger size and lower density otoconia were found in an animal model of osteoporosis [9]. Deficient serum vitamin D levels might be associated with recurrent BPPV, and vitamin D supplementation could reduce the risk of recurrence [10, 11]. In a pilot study, certain bone turnover biochemical markers, such as N-terminal propeptide of type 1 collagen (P1NP), were elevated in BPPV cases related to osteoporosis, while the serum vitamin D and serum Ca^2+^ levels were normal, consistent with the local effect of osteoporosis in the peripheral vestibular system [12]. Together, these findings may indicate that the imbalance between bone formation and bone resorption, which occurs with aging or during menopause, might disturb the local Ca^2+^ homeostasis of the inner ear, resulting in a high prevalence of BPPV in older patients and the risk of recurrence.

Dynamic changes in bone turnover can be better estimated by measuring various bone biochemical markers in the blood and urine, while bone mineral density (BMD) changes occur relatively slowly and may not signify the current rate of bone loss. Recent studies have established the potential utility of bone turnover markers (BTMs) to identify patients with rapid bone loss earlier, to make direct therapeutic decisions, and to monitor treatment responses. Because the maintenance of calcified otoconia requires specific interaction between the otoconial membrane and surrounding microenvironments, analyzing systemic Ca^2+^ metabolism in BPPV patients would be the first step to develop treatment strategies to reduce the incidence of recurrent BPPV or to predict those patients with a high risk of developing BPPV.

The aim of the present study was to evaluate the association between Ca^2+^ and vitamin D status and BPPV occurrence, as well as the status of bone biochemical markers in osteoporotic patients diagnosed with idiopathic BPPV.

## Materials and methods

### Subjects

The study enrolled a total of 132 postmenopausal women who were referred to the Vertigo Clinic and visited the CHA University Bundang Hospital between August 2008 and October 2013 and were then followed for at least one year. The data were retrospectively reviewed. The patients were diagnosed as canalolithiasis or cupulolithiasis in either the vertical canals or the horizontal canal, as established through typical positional nystagmus with no history of other vestibular diseases or head trauma. In addition, the following inclusion criteria were applied to all groups: [1] available BMD, BTMs and actual 25-hydroxy vitamin D (25(OH)D) values, which were measured within 3 months of the diagnosis for BPPV group, and [2] participants with no prior fractures, any systemic or chronic disease or current medications influencing their BMD or BTMs results, such as chronic renal failure, liver or bile duct disease and hormonal disorders. Positional nystagmus was evaluated using video electronystagmography (VNG). Posterior canal BPPV was diagnosed when Dix-Hallpike maneuvers generated upbeat-torsional, geotropic nystagmus. Horizontal canal BPPV was associated with distinctly horizontal nystagmus, which changes direction with head position changes (i.e., supine head turning). Anterior canal BPPV was diagnosed, with paroxysmal downbeating nystagmus, occasionally with a torsional component following Dix-Hallpike positioning, which was unrelated to any brainstem or cerebellar lesion. Recurrent BPPV was defined as two or more sporadic episodes of positional vertigo with a documented nystagmus free interval. The control group consisted of 52 osteoporotic women with no history of vertigo/dizziness who were selected from the Osteoporosis Clinic. The study was approved by the CHA University Bundang Hospital Ethics Committee. The informed consent was not obtained because the study was retrospectively performed on the basis of routine clinical data. All data were anonymized and de-identified prior to analysis.

### BMD and bone turnover marker measurements

Height and body weight were measured using standard methods with the patients wearing light clothes. Body mass index (BMI) was calculated as weight divided by height squared (kg/m^2^). A dual X-ray absorptiometry (DXA) scan (Discovery-W, Hologic Inc.) was obtained for all subjects. BMD (g/cm^2^) was measured at the lumbar spine (L1–L4) and femur. A T score, derived from the DXA measurement, expresses an individual’s BMD in standard deviations calculated from manufacturer-provided references. A diagnosis of osteopenia or osteoporosis was made using the World Health Organization (WHO) T-score criteria; a T score ≥ -1 is considered to indicate normal BMD; osteopenia was diagnosed with -2.5 < T score < -1; and osteoporosis was diagnosed with a T score ≤ -2.5.

The laboratory investigations included serum total calcium, phosphate, total cholesterol, low density lipoprotein (LDL) cholesterol, transaminase activities (GOT and GPT), creatinine and albumin, which was measured using automated standard laboratory methods. The serum ionized calcium level (iCa^2+^) was evaluated to identify calcium abnormalities when the serum total calcium (corrected for albumin) [13] was not within normal ranges. Actual 25(OH)D was measured through radioimmunoassay (CLIA, DiaSorin). A serum vitamin D deficiency was defined at 25(OH)D < 20 ng/mL [14]. Creatinine clearance was calculated from the plasma creatinine level [15]. Serum thyroid-stimulating hormone (TSH) and free T4 concentrations were also measured. The parathyroid hormone level was assessed in patients with abnormal serum calcium levels to determine the cause. The following bone turnover biochemical markers were analyzed: 1) bone resorption markers, including Carboxy-terminal telopeptide of type I collagen (s-CTX) (β-CrossLaps EIA, COBAS 6000, Roche diagnostics) and urinary free deoxypyridinoline (u-DPD) (EIA, Sunrise, Tecan), corrected for creatinine, and 2) bone formation markers, including serum total alkaline phosphatase (ALP) and serum osteocalcin (N-MID Osteocalcin EIA, COBAS 6000, Roche Diagnostics).

### Statistics

The data were analyzed using IBM SPSS Statistics Version 22 for Windows. Multiple means were compared among groups as indicators of discriminant validity through one-way ANOVA’s, followed by pair wise post hoc tests. For the post-hoc comparisons, we used Scheffe’s test. Pearson’s correlation coefficient was used to study the linear correlation between the variables. A chi-square test was applied to analyze the significance of the multiple comparison of relative frequencies among the groups. The odds-ratio (OR) was calculated, and multiple logistic regression analysis was used to estimate the odds ratios for the association between BPPV and the various factors. Test results with *P* < 0.05 were regarded as statistically significant.

## Results

### Clinical characteristics of the patients

The mean age of the total 132 patients was 63.0 ± 10.0 years (range, 49~81 years), and the mean age of the 52 control patients was 63.4 ± 9.0 years (range, 49~79 years). The mean follow-up time was 21.0 ± 12.7 months for the patients. The demographic and clinical characteristics of the patients and controls are summarized in Table 1. Based on the DXA results, the patients were divided into three groups: group 1, normal BMD (T score ≥ -1, n = 34); group 2, osteopenia (-2.5 < T score < -1, n = 40); and group 3, osteoporosis (T score ≤ -2.5, n = 58). There were no significant between-group differences in terms of age. The BMI results were similarly distributed in all groups, and 2 (5.9%) patients in group1, 1 (2.5%) patient in group 2, 3 (5.2%) patients in group3 and 2 (3.8%) patients in controls had low BMI below 18.5 kg/m^2^. Overall, there were no significant differences between the groups in terms of the presence of various risk factors, including hyperlipidemia, diabetes and hypertension. The history of smoking and current alcohol consumption were similar, regardless of the BMD results. All patients had normal thyroid hormone levels.

**Table 1:** Demographic data of the patients (N = 132) and controls (N = 52).

	BPPV Patients			Controls	
	Normal (N = 34)	Osteopenia (N = 40)	Osteoporosis (N = 58)	Osteoporosis (N = 52)	*P*
Age (years)	60.9 ± 10.0	61.4 ± 9.0	63.3 ± 9.0	63.4 ± 9.0	NS
BMI (kg/m^2^)	23.7 ± 3.9	23.9 ± 2.9	22.8 ± 2.9	22.9 ± 3.2	NS
Lumbar BMD (g/cm^2^)	0.982 ± 0.093	0.797 ± 0.075	0.649 ± 0.083	0.633 ± 0.102	
*T*-score	-0.2 ± 0.7	-1.8 ± 0.5	-3.1 ± 0.6	-3.2 ± 0.9	
Femur neck BMD (g/cm^2^)	0.786 ± 0.130	0.659 ± 0.092	0.543 ± 0.104	0.546 ± 0.125	
*T*-score	-0.2 ± 1.2	-1.4 ± 0.7	-2.5 ± 0.9	-2.4 ± 1.1	
Smoking (%)	2 (5.9)	2 (5.0)	3 (5.2)	4 (7.7)	NS
Alcohol (%)	3 (8.8)	4 (10.0)	6 (10.3)	6 (11.5)	NS
Hyperlipidemia (%)	7 (20.6)	8 (20.0)	11 (19.0)	13 (25.0)	NS
Diabetes (%)	7 (20.6)	9 (22.5)	12 (20.7)	13 (25.0)	NS
Hypertension (%)	12 (35.3)	14 (35.0)	20 (34.5)	20 (38.5)	NS

Data are presented as the mean ± SD. Significant differences are marked, NS, not significant.

BMI, body mass index; BMD, bone mineral density

### Calcium, vitamin D and bone turnover markers

The biochemical characteristics of each group are presented in Table 2. In all groups, the mean serum calcium, phosphate, and albumin levels and creatinine clearance were within the standard laboratory reference ranges, and there were no significant between-group differences. Low serum calcium levels corrected for albumin binding were found in 2 (5.9%) patients in group 1, 2 (5.0%) patients in group 2, 4 (6.9%) patients in group 3 and 3 (5.8%) control patients, with no significant differences among the groups. Hypocalcemia was mild for these patients, with a mean iCa^2+^ level of 1.07 ± 0.02 mM/L. The parathyroid hormone levels (intact PTH) for these patients were within the standard laboratory reference range (32.6 ± 7.1 pg/ml).

**Table 2:** Biochemical parameters.

	BPPV Patients									Controls			
	Normal			Osteopenia			Osteoporosis			Osteoporosis			*P*
Calcium (mg/dL)	9.1	±	0.5	9.2	±	0.5	9.0	±	0.6	9.0	±	0.5	NS
Phosphate (mg/dL)	3.6	±	0.6	3.7	±	0.5	3.6	±	0.7	3.6	±	0.7	NS
Creatinine clearance (ml/min)	80.3	±	9.2	79.0	±	8.1	78.1	±	8.3	77.8	±	9.4	NS
25-(OH) vitamin D (ng/mL)	34.2	±	14.3	35.7	±	15.3	22.3	±	12.7**	30.3	±	18.6	.005
ALP (IU/L)	173.8	±	38.6	174.8	±	42.1	181.2	±	66.7	182.5	±	42.5	NS
Osteocalcin (ng/mL)	9.2	±	6.3	13.0	±	9.1*	17.1	±	11.3*	16.1	±	10.7*	.035
s-CTX (ng/mL)	0.48	±	0.47	0.38	±	0.32	0.37	±	0.31	0.44	±	0.57	NS
u-DPD (nM/mM creatinine)	6.8	±	4.9	6.7	±	2.5	12.3	±	5.1*	8.7	±	5.9	.04

Data are presented as the mean score ± SD.

Significant differences are marked

*P < .05

**P < .01; NS, not significant.

ALP, total alkaline phosphatase; s-CTX, serum Carboxy-terminal telopeptide of type I collagen; u-DPD, urinary free deoxypyridinoline

The BMD, *T*-score appeared to be directly related to age and older subjects tended to have lower T scores (*r* = -0.31, *P* < 0.001). Among all BTMs used in the present study, osteocalcin was moderately correlated with BMD, *T*-score (*r* = -0.30, *P* < 0.05). Other bone turnover markers such as ALP, u-DPD and s-CTX did not correlate well with the underlying BMD, *T*-score.

The mean level of u-DPD was significantly elevated in the osteoporotic patients with BPPV (Table 2). The mean level of osteocalcin was also statistically higher in the patients with reduced BMD than the patients with normal BMD. However, the analysis of the serum ALP and s-CTX regarding BMD *T*-score did not demonstrate any significant differences among three groups.

**Fig 1:**
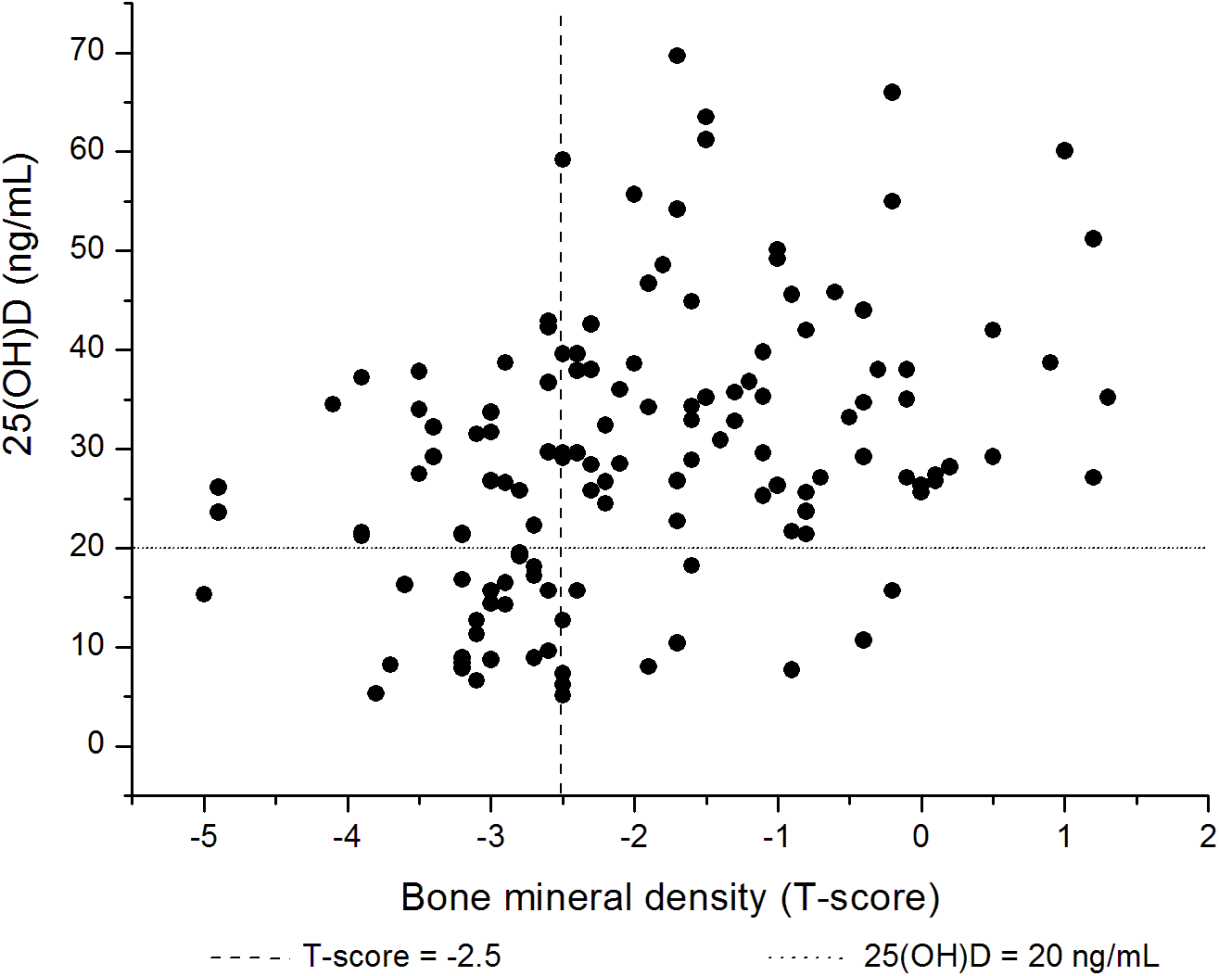
Relationship between 25(OH)D and bone mineral density (T-score) in patients with BPPV.

The prevalence of vitamin D deficiency was 11.8% (4/34) in group 1, 15% (6/40) in group 2 and 43.1% (25/58) in group 3, respectively. The proportion of vitamin D deficiency was significantly higher in group 3, compared to other patients (*P* < 0.01, OR = 4.8). There was a moderate, significant positive correlation between the 25(OH)D and BMD results in the patients with BPPV, after adjusting for age (*r* = 0.47, *P* < 0.01, Fig 1). However, there was no significant correlation between the 25(OH)D level and the BTMs. BPPV was recurrent in 46 patients (34.8%) during the follow-up. Multiple logistic regression analyses were used to evaluate the connection between BPPV and associated variables including age, osteopenia, osteoporosis, and serum vitamin D deficiency (Table 3). The regression analyses demonstrated that osteoporosis and vitamin D deficiency were risk factors for BPPV.

**Table 3:** Association between osteoporosis and benign paroxysmal positional vertigo, according to the multiple logistic regression analysis.

	OR	*P* value	95% CI
Age	1.1	> .05	0.95, 1.04
Bone mineral density			
Osteopenia (-2.5 < T score < -1)	1.7	> .05	0.79, 6.27
Osteoporosis (T score ≤ -2.5)	2.8	0.02	1.21, 9.40
Vitamin D deficiency (25(OH)D < 20 ng/mL)	2.0	0.02	0.93, 1.03

OR, odds ratio; CI, confidence interval

## Discussion

The most salient findings of this study are 1) osteoporosis is associated with idiopathic BPPV in postmenopausal women, 2) higher bone turn-over rates are present in osteoporotic patients who are diagnosed with idiopathic BPPV, and 3) vitamin D deficiency is highly prevalent in approximately half of osteoporotic patients with idiopathic BPPV, which could adversely affect calcium metabolism in the vestibule.

Osteoporosis and osteopenia are characterized by reduced bone mass, increased bone turnover, and increased susceptibility to fracture; the diseases, which are common complications of aging, are becoming a major public health concern due to the aging of the global population. The reported prevalence of osteoporosis in women older than 50 years of age varies from 7.9% to 22.6%, depending on the study population and adoptive reference standards [16, 17]. In Korea, the prevalence of osteoporosis at the lumbar spine in female patients over age 50 was 24.0~28.6% [18, 19].

There has been growing evidence that osteoporosis is linked to the incidence or recurrence of BPPV. First, osteopenia and osteoporosis were more prevalent in middle-aged and elderly subjects, particularly postmenopausal women. The idiopathic BPPV patients who were over 50 years of age demonstrated significantly reduced BMD values approximately 50~80% of cases [7, 8, 11, 20]. The prevalence of osteoporosis for these patients has been reported up to 47%, which was significantly higher than expected. A high prevalence of osteoporosis (43.9% [58/132]) was also observed in the present study. In addition, the prevalence of undiagnosed BPPV was approximately 31% among the patients with a history of osteopenia or osteoporosis; this rate was 3 times higher than the expected rate of unrecognized BPPV in a geriatric population 70 years of age and older [3, 11]. A significant negative association between the incidence of BPPV and treated osteoporosis has been reported [21].

Second, the recurrence rate of BPPV was significantly higher in the patients with reduced BMD. An increased recurrence rate was observed in approximately 57% of the osteoporotic patients who were diagnosed with BPPV, while 16.1% of the patients with normal BMD experienced a recurrence during the 1-year follow-up period. In addition, multiple canalith repositioning procedures were required for symptom cessation in the patients with osteopenia/osteoporosis who were diagnosed with BPPV [20, 22].

Otoconia, CaCO_3_ bio-minerals precipitated around a proteinous core, are embedded in otoconial complexes, which are composed of the subcupular meshwork and amorphous gelatinous membranes [23]. Normally, otoconia are already matured shortly after birth and undergo maintenance thereafter. However, the mechanisms of otoconial formation and maintenance are only partially understood. Otoconia have unique spatial specificity overlying the macular epithelium, and they require specific Ca^2+^ sequestration within the otoconial complexes for crystal seeding, due to the inherent nature of low Ca^2+^ micro-environment in the inner ear. An extraordinary low Ca^2+^ concentration of endolymph (vestibule, ~280 μM) has been known to dissolve dislodged otoconia and to contribute to spontaneous recovery of BPPV [24, 25]. The ability to dissolve otoconia was significantly compromised in vitro due to elevated endolymphatic Ca^2+^ content [25]. Although mature otoconia have slow Ca^2+^ turn-over rates [26], they appear to be influenced by various factors, which means that otoconia might play a role as Ca^2+^ ion reservoirs to maintain local Ca^2+^ homeostasis [27, 28]. Exposure to ototoxic drugs, such as streptomycin, resulted in the formation of abnormal giant otoconia [29]. In addition, distinctive changes in the morphometry and distributional pattern of otoconia have been commonly observed in aged rats and in an ovariectomized rat model of osteoporosis [9, 30, 31]. These findings might suggest that age-dependent degeneration and reduced otoconia volume, which is also a serious risk factor for loss of balance in elderly patients, could be adversely affected by metabolic bone diseases, such as osteoporosis. Measuring bone turn-over rates at a systemic level might be the first step in providing insight into the pathogenesis of idiopathic BPPV.

BMD, as assessed by DXA scans, remains the gold standard for diagnosing osteoporosis. However, the current status of bone strength is not assessed by either BMD or clinical risk factors but rather the rate of bone remodeling. BMD could not be an early indicator of pathological changes in the bones or a prognostic measure of the risk of fractures. Treatment-related changes in BMD cannot be reliably detected for 1~2 years, whereas dynamic changes in bone turnover, as estimated by BTMs in the blood and urine, can account for most treatment responses after 3 months [32]. Higher bone turnover rates are generally associated with accelerated bone loss and potential deterioration in bone quality, with an increased risk of fracture, although challenges include great biological and individual variability of BTMs. In various studies, the mean urinary excretion or serum level of individual BTMs has been higher (to varying degrees) in patients with osteoporosis than in healthy subjects [33–35]. However, these results are often not consistent, and the values of healthy subjects and patients with osteoporosis overlap substantially. In the present study, the levels of different BTMs were not well correlated with BMD *T*-scores in patients who were diagnosed with idiopathic BPPV. However, there appears to be an overall increase of bone turnover in osteoporotic patients with BPPV because the mean serum osteocalcin and urinary DPD levels were elevated in the osteoporotic patients with BPPV compared with the patients with normal BMD or the controls. In addition, vitamin D deficiency was commonly present in the osteoporotic patients with BPPV, even in the patients whose serum calcium levels were normal. Vitamin D adequacy is essential for optimal Ca^2+^ metabolism, although there is controversy about the optimum level of 25(OH)D. However, even higher vitamin D levels appear to be required for specific conditions, such as osteoporosis, to improve treatment outcomes [36]. Vitamin D receptor deficient (VDR^-/-^) mice are known to be associated with decreased balance function [37], and several studies have proposed an inverse relationship between low serum vitamin D levels and recurrent BPPV [10, 11].

The present study has some limitations. Overall, use of the clinical effectiveness of BTMs to address diagnostic, prognostic and therapeutic decisions is still limited, in part because of the lack of robust data concerning the comparative predictive values of the different BTMs. The biological levels of BTMs have also been known to vary throughout the various stages of fracture healing, which could be dependent upon an individual host’s metabolism. The present study was also hindered by the lack of an appropriate control group without osteoporosis. Consequently, it is currently impossible to draw concrete conclusions as to whether BTMs are able to independently identify or predict BPPV risk in patients with osteoporosis. However, the present study suggests that osteoporotic patients with BPPV are negatively influenced by Ca^2+^ metabolism, along with vitamin D deficiency, which may be a risk factor for BPPV. Further studies are needed to clarify the contribution of Ca^2+^ metabolism and BTMs to BPPV risks and their interactions with otoconial complexes.

## Conclusion

In conclusion, the present study demonstrates that the prevalence of BPPV in osteoporotic patients is associated with vitamin D deficiency and high bone turnover rates at the systemic level, which could disturb local Ca^2+^ homeostasis as well as the architecture of otoconial complexes in the inner ear. It is conceivable that dysfunctional Ca^2+^ metabolism might represent a significant proportion of idiopathic cases of BPPV in postmenopausal women.

## Supporting information

S1 Dataset (XLS)
